# The effect of anticancer treatment on cancer patients with COVID‐19: A systematic review and meta‐analysis

**DOI:** 10.1002/cam4.3692

**Published:** 2020-12-31

**Authors:** Hanqing Liu, Dan Yang, Xinyue Chen, Zhihong Sun, Yutong Zou, Chuang Chen, Shengrong Sun

**Affiliations:** ^1^ Department of Thyroid and Breast Surgery Renmin Hospital of Wuhan University Wuhan Hubei PR China; ^2^ Department of Cardiology Renmin Hospital of Wuhan University Wuhan Hubei PR China; ^3^ Department of Laboratory Medicine Peking Union Medical College Hospital Chinese Academy of Medical Sciences Dongcheng District Beijing PR China

**Keywords:** cancer, chemotherapy, COVID‐19, hematological malignancy

## Abstract

**Background:**

The relationship between cancer and COVID‐19 has been revealed during the pandemic. Some anticancer treatments have been reported to have negative influences on COVID‐19‐infected patients while other studies did not support this hypothesis.

**Methods:**

A literature search was conducted in WOS, PubMed, Embase, Cochrane Library, CNKI and VIP between Dec 1, 2019 and Sept 23, 2020 for studies on anticancer treatments in patients with COVID‐19. Cohort studies involving over 20 patients with cancer were included. The characteristics of the patients and studies, treatment types, mortality, and other additional outcomes were extracted and pooled for synthesis. RRs and forest plots were adopted to present the results. The literature quality and publication bias were assessed using NOS and Egger's test, respectively.

**Results:**

We analyzed the data from 29 studies, with 5121 cancer patients with COVID‐19 meeting the inclusion criteria. There were no significant differences in mortality between patients receiving anticancer treatment and those not (RR 1.17, 95%CI: 0.96–1.43, *I^2^*=66%, *p* = 0.12). Importantly, in patients with hematological malignancies, chemotherapy could markedly increase the mortality (RR 2.68, 95% CI: 1.90–3.78, *I^2^*=0%, *p* < 0.00001). In patients with solid tumors, no significant differences in mortality were observed (RR 1.16, 95% CI: 0.57–2.36, *I^2^*=72%, *p* = 0.67). In addition, our analysis revealed that anticancer therapies had no effects on the ICU admission rate (RR 0.87, 95% CI: 0.70–1.09, *I^2^*=25%, *p* = 0.23), the severe rate (RR 1.04, 95% CI: 0.95–1.13, *I^2^*=31%, *p* = 0.42), or respiratory support rate (RR 0.92, 95% CI: 0.70–1.21, *I^2^*=32%, *p* = 0.55) in COVID‐19‐infected patients with cancer. Notably, patients receiving surgery had a higher rate of respiratory support than those without any antitumor treatment (RR 1.87, 95%CI: 1.02–3.46, *I^2^*=0%, *p* = 0.04).

**Conclusions:**

No significant difference was seen in any anticancer treatments in the solid tumor subgroup. Chemotherapy, however, will lead to higher mortality in patients with hematological malignancies. Multicenter, prospective studies are needed to re‐evaluate the results.

## Graphical abstract

No significant difference was seen in any anticancer treatments in solid tumor subgroup. Chemotherapy, however, will lead to a higher mortality in patients with hematological malignancies.

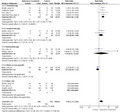

## 1 INTRODUCTION

The sudden outbreak and worldwide epidemic of coronavirus disease 2019 (COVID‐19) have brought great challenges and heavy burdens to global public health since December 2019. To date, the world has been fighting against this deadly disease, which is caused by a novel coronavirus known as severe acute respiratory syndrome‐related coronavirus 2 (SARS‐CoV‐2).^1^ Globally, the number of people who are infected with COVID‐19 is dramatically increasing every day. As of July 24, 2020, there had been more than 38 million confirmed cases and over 1,090,000 deaths in 235 countries, areas or territories around the world.^2^

Notably, up‐to‐date reports suggest that every year there are approximately 18.1 million new patients with cancer in the world.^3^ A growing number of studies have revealed that during the pandemic, patients with cancer have a higher risk of developing COVID‐19 and COVID‐19‐related complications.^4^, ^5^, ^6^ Patients with cancer also exhibit severe conditions and poor prognosis when diagnosed with COVID‐19.^7^ Patients with cancer are usually in severe immunosuppressive states, which is caused by the cancer itself and the anticancer treatments. In addition, patients suffering from cancer regularly visit medical facilities for anticancer treatment or monitoring, which results in an increased chance of contact with a source the virus.

It is well recognized that patients with cancer require individualized anticancer treatment, such as surgery, chemotherapy, immunotherapy, radiotherapy, and targeted treatment. Standard anticancer therapies can effectively enhance the quality of life and improve the prognosis of patients with cancer. However, emerging studies suggest that COVID‐19‐infected cancer patients receiving systematic anticancer therapy are at a higher risk than those who receive no antitumor treatment,^8^ especially hematological patients receiving chemotherapy.^9^ Interestingly, there are also clinical studies strongly, indicating that anticancer treatments have no deteriorating effects on clinical outcomes.^10^, ^11^ Thus, whether COVID‐19‐infected cancer patients with versus without anticancer treatments have a higher risk of unfavorable clinical outcomes remains unclear. Therefore, by performing a systematic review and meta‐analysis of the emerging studies, we aimed to qualify the potential effects of anticancer therapies on the clinical outcomes, such as mortality, admission to the intensive care unit (ICU), and the severity of COVID‐19, in patients with cancer infected with COVID‐19. We hope that our findings will provide information to oncologists or other physicians for the appropriate management of patients with cancer infected with COVID‐19 during the pandemic.

## 2 Methods

### 2.1 Study protocol

We planned, conducted, and reported the systematic review and meta‐analysis in accordance with the Preferred Reporting Items for Systematic Review and Meta‐Analysis Protocols (PRISMA‐P) 2015 Statement (Supplement 1).^12^ The whole protocol has been registered in the PROSPERO database (CRD42020200736).

### 2.2 Literature search

Given that many early studies were conducted by Chinese researchers, both English and Chinese databases were searched to minimize language bias. The searched English databases included Web of Science (WOS), PubMed, Embase, and Cochrane Library, while the Chinese databases included the China National Knowledge Infrastructure (CNKI) and the China Science and Technology Journal Database (VIP). One researcher (HQ L) with meta‐analysis experience drafted the search strategy, which was revised and approved by other researchers. The following medical subject headings (MeSH) and non‐MeSH keywords were arranged in the search sentence: (COVID‐19 OR SARS‐CoV‐2 OR 2019‐nCoV OR coronavirus) AND (tumor OR carcinoma OR cancer OR hematolog* OR haematolog* OR leukemia OR lymphoma OR myeloma) (Table 1). The published dates of studies were limited to Dec‐01, 2020 to Sept‐23, 2020, with no restriction on language. The lists of references were screened to identify any missed studies. The literature from different sources was then imported into Endnote (version X9.0) for duplicate exclusion.

**TABLE 1:** Medical terms for literature search

Language	Keyword 1	Keyword 2
English	COVID−19	tumor
	SARS‐CoV−2	carcinoma
	2019‐nCoV	cancer
	coronavirus	hematolog*
		haematolog*
		leukemia
		lymphoma
		myeloma
Chinese	新冠肺炎	肿瘤
	冠状肺炎	癌症
		血液
		淋巴瘤
		白血病

The Boolean operator “AND” was placed between different keyword group while “OR” was placed within the terms of same group.

### 2.3 Study selection and definition

In this systematic review, any research articles meeting the following criteria were included for the further data extraction and synthesis: (a) studies reporting the effects of any antitumor treatments on mortality, ICU admission rate, rate of respiratory support or severe/critical rate in patients with cancer diagnosed with COVID‐19; (b) patients ≥18 years old, and (c) the relative risk (RR) can be extracted or relevant statistics are provided for calculation.

Studies meeting the following criteria were excluded: (a) review, news, editorial, comment, guideline, clinical experience, basic research, study protocol or case report; (b) cancer patients <20 or cannot be separated from non‐cancer patients; (c) patients were diagnosed with other viral pneumonia, such as SARS or MERS and (d) data derived from the same group of patients.

Two independent reviewers (HQ L and D Y) carried out the literature screening with blindness to each other. The titles and abstracts were screened in the first two rounds for efficiency. Then full articles were obtained for subsequent selection according to the criteria. Disagreements were resolved via consultation with a senior reviewer (C C).

The diagnosis of COVID‐19 should be based on RT‐PCR or antibody tests. Due to the changing standard for diagnosis, the shortage of testing kits in some regions, and the unsatisfactory accuracy of laboratory tests,^13^, ^14^ a CT finding or a consensus based on symptoms by ≥2 skillful physicians was also acceptable. No restriction was cast on cancer types, but cancer needed to be concurrent with COVID‐19, and a cancer history was obviously unacceptable. Any type of antitumor treatment should be administered within 3 months before the diagnosis of COVID‐19. The end‐points should be measured in hospitals or medical institutions. Respiratory support was defined as mechanical ventilation, facial mask or any other mechanical technique improving the respiratory function. The definition of the severe/critical rate should conform with the Diagnosis and Treatment Protocol for Novel Coronavirus Pneumonia released by the National Health Commission,^15^ with no limitation on its version.

### 2.4 Data extraction and quality assessment

Two authors (HQ L and D Y) extracted the data from the included studies independently and then cross‐checked their results. Disagreements were resolved via consensus or consultation with a senior reviewer (C C). The following data were collected in a worksheet: first author, published date, country, study design, number of patients, number of females, median age, comorbidities, detection of COVID‐19, cancer type, interpretation type, and outcome. The relative risks (RRs) were obtained from the papers or calculated based on original statistics.

The Newcastle‐Ottawa Quality Assessment Scale for Cohort Studies was adopted in the quality assessment^16^ (Supplement 2). Eight questions in the scale were arranged into three groups: patient selection, comparability and outcome reliability. Two reviewers (XY C and ZH S) assessed the risk of bias independently with blindness to each other. Disagreements were settled by a third reviewer (YT Z).

### 2.5 Data synthesis and statistical analysis

The data synthesis was performed on RevMan (version 5.3) and the publication bias was calculated with Stata (version 15.1). Relative risks and 95% confidence intervals (CIs) were calculated to compare the mortality rate and other additional outcomes between patients receiving antitumor treatments or not. A *p*‐value <0.05 was deemed statistically significant. The inconsistency index (*I^2^* statistic) and Cochran's *Q* test were adopted in the assessment of heterogeneity. The 50% *I^2^* was defined as a cut‐off for low and high heterogeneity. With low heterogeneity, a fixed‐effects model was used to estimate the average effect and its precision. If the heterogeneity was high, a random model was adopted. Subgroup analyses were then performed on specific antitumor treatments and different cancer types (solid tumor or hematological malignancy). The minimum number of articles for data synthesis was two in each group. The funnel‐plot asymmetry designed by Egger et al.^17^ was employed to estimate the publication bias.

## 3 RESULTS

### 3.1 Search results

**FIGURE 1:**
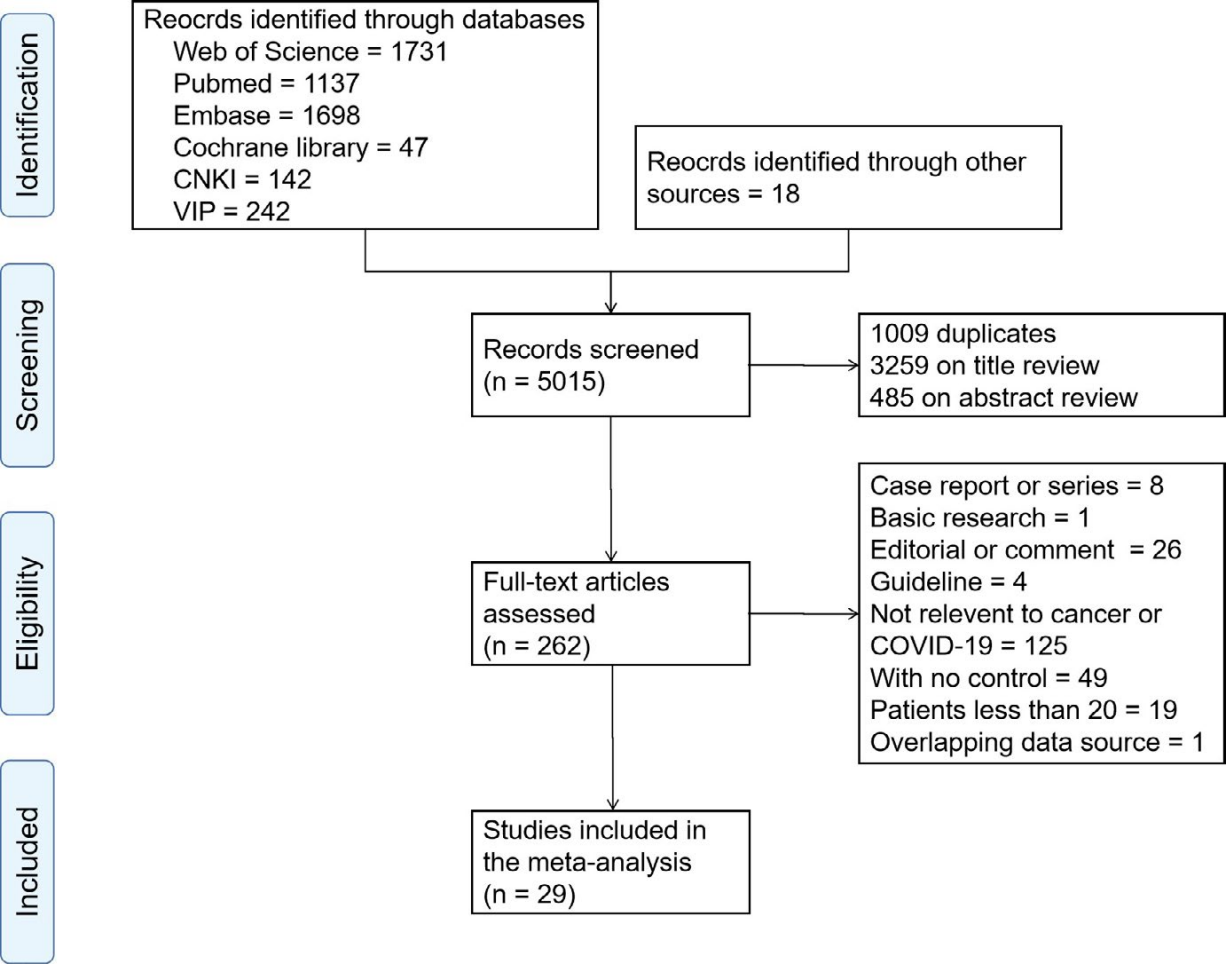
Flow chart of study selection

A total of 5015 records were identified in our initial search. Of these, 1009 papers were duplicates and thus excluded. After review of titles and abstracts, 3744 papers that did not fulfill our criteria for full‐text review were removed, leaving 262 papers for further evaluation. Another 233 papers were excluded because they were case reports/series, basic studies, editorials, comments, guidelines, articles that were not relevant to cancer/COVID‐19, articles with no control group, articles with fewer than 20 patients, or overlapping data sources. Eventually, 29 studies were included in our systematic review and meta‐analysis (Figure 1).

### 3.2 Study characteristics

A total of 5121 patients with cancer in 29 studies were included in this meta‐analysis.^6^, ^7^, ^8^, ^9^, ^10^, ^11^, ^40^ The characteristics of the studies included in this meta‐analysis are presented in Table 2. Of the remaining 29 studies, eight were conducted in China, six in the United States, four in the United Kingdom, three in Spain, two in France, two in Italy, and four was performed in multiple countries. Patients with COVID‐19 were mainly confirmed by real‐time reverse transcriptase‐polymerase chain reaction (RT‐PCR). The sample sizes of the included studies ranged between 25 and 928, and the NOS scores varied from 5 to 7 (Supplement 3).

**TABLE 2:** Characteristic of included studies and patient population

Author	Country	Published date	Type of study	Number of patients	Female (%)	Median age (years)	Type of cancer	Anticancer treatment	Outcomes	Diagnosis method for COVID−19
Assaad et al^18^	France	2020/06/07	retrospective Single‐center	55	29(52.7)	64	non‐specific	chemotherapy targeted therapy	mortality rate	RT‐PCR
Booth et al^19^	UK	2020/06/16	prospective multicenter	66	25(37.9)	73	hematological malignancies	chemotherapy targeted therapy	mortality rate, respiratory support	RT‐PCR, CT, and clinical features
Cattaneo et al^20^	Italy	2020/09	retrospective multicenter	102	36(35.3)	68	hematological malignancies	chemotherapy	mortality rate	RT‐PCR
Dai et al^8^	China	2020/04/28	retrospective multicenter	105	48(45.7)	64	non‐specific	surgery chemotherapy immunotherapy targeted therapy radiotherapy	mortality rate respiratory support ICU admission rate severe/critical rate	RT‐PCR
Fox et al^11^	UK	2020/07/12	retrospective single center	55	17(31.0)	63	hematological malignancies	chemotherapy immunotherapy	mortality rate severe/critical rate	RT‐PCR, CT, and clinical features
Jee et al^21^	US	2020/08/15	retrospective single‐center	309	150(48.5)	NA	non‐specific	chemotherapy immunotherapy targeted therapy	severe/critical rate	RT‐PCR
Kuderer et al^22^	US, Canada, and Spain	2020/05/28	ambispective multicenter	928	459(49.5)	66	non‐specific	surgery and chemotherapy	mortality rate ICU admission rate respiratory support	RT‐PCR
Lara et al^23^	US	2020/07/30	retrospective multicenter	121	NA	64	gynecologic cancer	chemotherapy hormone therapy immunotherapy radiotherapy surgery targeted therapy	severe/critical rate	RT‐PCR and CT
Lee et al^10^	UK	2020/05/28	prospective multicenter	800	349(43.6)	69	non‐specific	chemotherapy hormone therapy immunotherapy radiotherapy surgery targeted therapy	mortality rate	RT‐PCR
Liu et al^24^	China	2020/09/15	retrospective multicenter	216	103(47.7)	63	solid tumor	antitumor therapy	mortality rate	RT‐PCR
Luo et al^25^	US	2020/07/23	retrospective single‐center	102	53(52.0)	68	lung cancer	chemotherapy targeted therapy immunotherapy	mortality rate ICU admission rate	RT‐PCR
Ma et al^26^	China	2020/05/14	retrospective single‐center	37	17(45.9)	62	solid tumor	antitumor therapy	severe/critical rate	RT‐PCR and/or antibody test
Mato et al^27^	International	2020/07/20	retrospective multicenter	198	73(36.9)	63	chronic lymphocytic leukemia	non‐specific	mortality rate	RT‐PCR
Mehta et al^28^	US	2020/05/01	retrospective single‐center	218	91(41.7)	69	non‐specific	chemotherapy immunotherapy radiotherapy	mortality rate	RT‐PCR
Pinato et al^29^	Italy, Spain and UK	2020/07	retrospective multicenter	204	77(37.7)	69	non‐specific	chemotherapy	mortality rate	RT‐PCR
Robilotti et al^30^	US	2020/06	retrospective single‐center	423	211(49.9)	NA	non‐specific	surgery chemotherapy	severe/critical rate	RT‐PCR
Rogado et al^31^	Spain	2020/05	retrospective single‐center	45	15(33.3)	71	solid tumor	chemotherapy	mortality rate	RT‐PCR
Russell et al^32^	UK	2020/07/22	ambispective single‐center	156	66(42.3)	65	non‐specific	chemotherapy targeted therapy immunotherapy	severe/critical rate	RT‐PCR
Sanchez‐Pina et al^9^	Spain	2020/08/14	prospective, single‐center	39	16(41.0)	65	hematological malignancies	chemotherapy targeted therapy	mortality rate	RT‐PCR
Scarfò et al^33^	International	2020/07/09	retrospective multicenter	190	64(33.7)	72	chronic lymphocytic leukemia	non‐specific	severe/critical rate	RT‐PCR
Stroppa et al^34^	Italy	2020/05/14	retrospective single‐center	25	5(20.0)	72	non‐specific	chemotherapy immunotherapy	mortality rate	RT‐PCR
Tian et al^6^	China	2020/05/29	retrospective multicenter	232	113(48.7)	64	non‐specific	surgery chemotherapy radiotherapy targeted therapy immunotherapy	severe/critical rate	RT‐PCR
Vuagnat et al^35^	France	2020/05/28	prospective, single‐center	58	NA	58	breast cancer	chemotherapy targeted therapy endocrine therapy	severe/critical rate	RT‐PCR and/or CT
Wang et la^36^	US	2020/07/14	retrospective single‐center	36	13(36.1)	67	multiple myeloma	immunotherapy	mortality rate	RT‐PCR
Yang et al^37^	China	2020/06	retrospective single‐center	52	24(46.2)	63	solid tumor	surgery chemotherapy immunotherapy	severe/critical rate	RT‐PCR
Yang et al^38^	China	2020/05/29	retrospective multicenter	205	109(53.2)	63	non‐specific	surgery chemotherapy radiotherapy targeted therapy immunotherapy	mortality rate	RT‐PCR
Yarza et al^39^	Spain	2020/06/06	prospective, single‐center	63	29(46.0)	66	solid tumor	chemotherapy endocrine therapy targeted therapy immunotherapy	severe/critical rate	RT‐PCR and/or radiology
Zhang et al^40^	China	2020/06	retrospective multicenter	107	47(43.9)	66	non‐specific	chemotherapy targeted therapy immunotherapy	severe/critical rate	RT‐PCR and/or radiology
Zhang et al^7^	China	2020/03/26	retrospective multicenter	28	11(39.3)	65	solid tumor	chemotherapy surgery radiotherapy target therapy immunotherapy	severe/critical rate	RT‐PCR

### 3.3 The effects of anticancer treatments on the outcomes of COVID‐19‐infected cancer patients

In the 29 included studies, anticancer therapies involved chemotherapy, surgery, targeted therapy, immunotherapy, radiotherapy, endocrine therapy, and other unspecific treatments. The outcomes evaluated included mortality, ICU admission rate, severe/critical rate, and the rate of respiratory support. In the current meta‐analysis, we aimed to evaluate the effects of various anticancer treatments on the outcomes of cancer patients infected with COVID‐19. No significant publication bias was found by either Egger test or the funnel test (*p* = 0.645) (Supplement 4).

The most common type of anticancer treatment among COVID‐19‐infected patients with cancer was chemotherapy (pooled rate of 30%, 95% CI: 23%‐39%) (*n* = 1478), followed by targeted therapy (pooled rate of 11%, 95% CI: 8%‐15%) (*n* = 263), radiotherapy (pooled rate of 10%, 95% CI: 7%‐15%) (*n* = 168), endocrine therapy (pooled rate of 9%, 95% CI: 4%‐20%) (*n* = 107), surgery (pooled rate of 8%, 95% CI: 5%‐13%) (*n* = 321), and immunotherapy (pooled rate of 8%, 95% CI: 6%‐10%) (*n* = 158). Fourteen studies reported severe/critical rates in patients with cancer infected with COVID‐19, with a pooled rate of 39% (95% CI: 26%‐59%) (*n* = 756). Seventeen studies provided data on mortality, and the pooled mortality rate was 27% (95% CI: 22%‐35%) (*n* = 817). Moreover, the pooled rates of ICU admission and respiratory support were 21% (95% CI: 13%‐33%) (*n* = 186) and 19% (95% CI: 9%‐40%) (*n* = 153), respectively. Additionally, 12 studies focused on solid tumors, and the pooled rate was 71% (95% CI: 70%‐72%) (*n* = 2517), in contrast, the pooled rate of hematological malignancies was 17% (95% CI: 16%‐17%) (*n* = 716).

**FIGURE 2:**
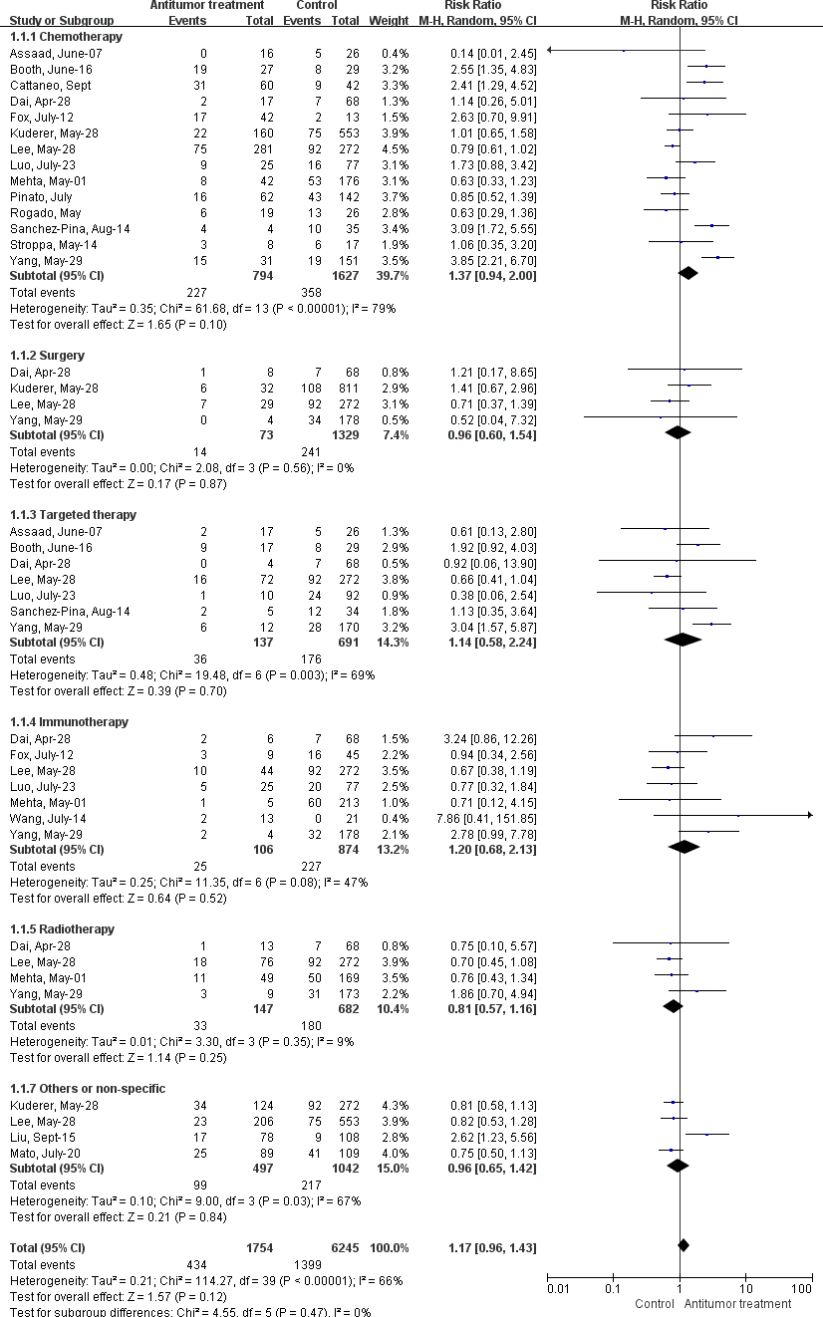
Forest plot for the association between antitumor treatments and risk of mortality in cancer patients with COVID‐19 using random‐effects model

Almost all the studies reported the mortality of patients with cancer infected with COVID‐19 (Figure 2). Fourteen studies provided data on the mortality of patients receiving chemotherapy. There were no significant differences between the chemotherapy group and the control group (RR 1.37, 95%CI: 0.94–2.00, *I^2^*=79%, *p* = 0.10). In addition, four studies focused on the mortality associated with surgery treatment, and data analysis revealed that no significant differences existed in patients with cancer receiving surgery or not (RR 0.96, 95% CI: 0.60–1.54, *I^2^*=0%, *p* = 0.87). Seven studies provided data on the effects of targeted therapy on patient mortality, and the analysis revealed that there were no significant differences in the targeted therapy group and control groups (RR 1.14, 95% CI: 0.58–2.24, *I^2^*=69%, *p* = 0.70). In addition, no changes in mortality were observed in patients receiving immunotherapy (RR 1.20, 95% CI: 0.68–2.13, *I^2^*=47%, *p* = 0.52), radiotherapy (RR 0.81, 95%CI: 0.57–1.16, *I^2^*=9%, *p* = 0.25) or others (RR 0.96, 95% CI: 0.65–1.42, *I^2^*=67%, *p* = 0.84) compared with those receiving no antitumor therapy

The ICU admission rate was another essential outcome and was related to the prognosis of patients (Supplement 5 Figure S1). In patients with cancer infected with COVID‐19, data analysis showed that patients receiving chemotherapy (RR 0.86, 95% CI: 0.61–1.22, *I^2^*=63%, *p* = 0.40), surgery (RR 1.45, 95% CI: 0.79–2.64, *I^2^*=0%, *p* = 0.23), targeted therapy (RR 1.33, 95% CI: 0.66–2.67, *I^2^*=0%, *p* = 0.43), immunotherapy (RR 0.94, 95%CI: 0.51–1.74, *I^2^*=0%, *p* = 0.84), or other anticancer treatments (RR 0.68, 95% CI: 0.45–1.03, *I^2^*=0%, *p* = 0.07) presented a similar rate of ICU admission compared to those who received no anticancer therapy.

**FIGURE 3:**
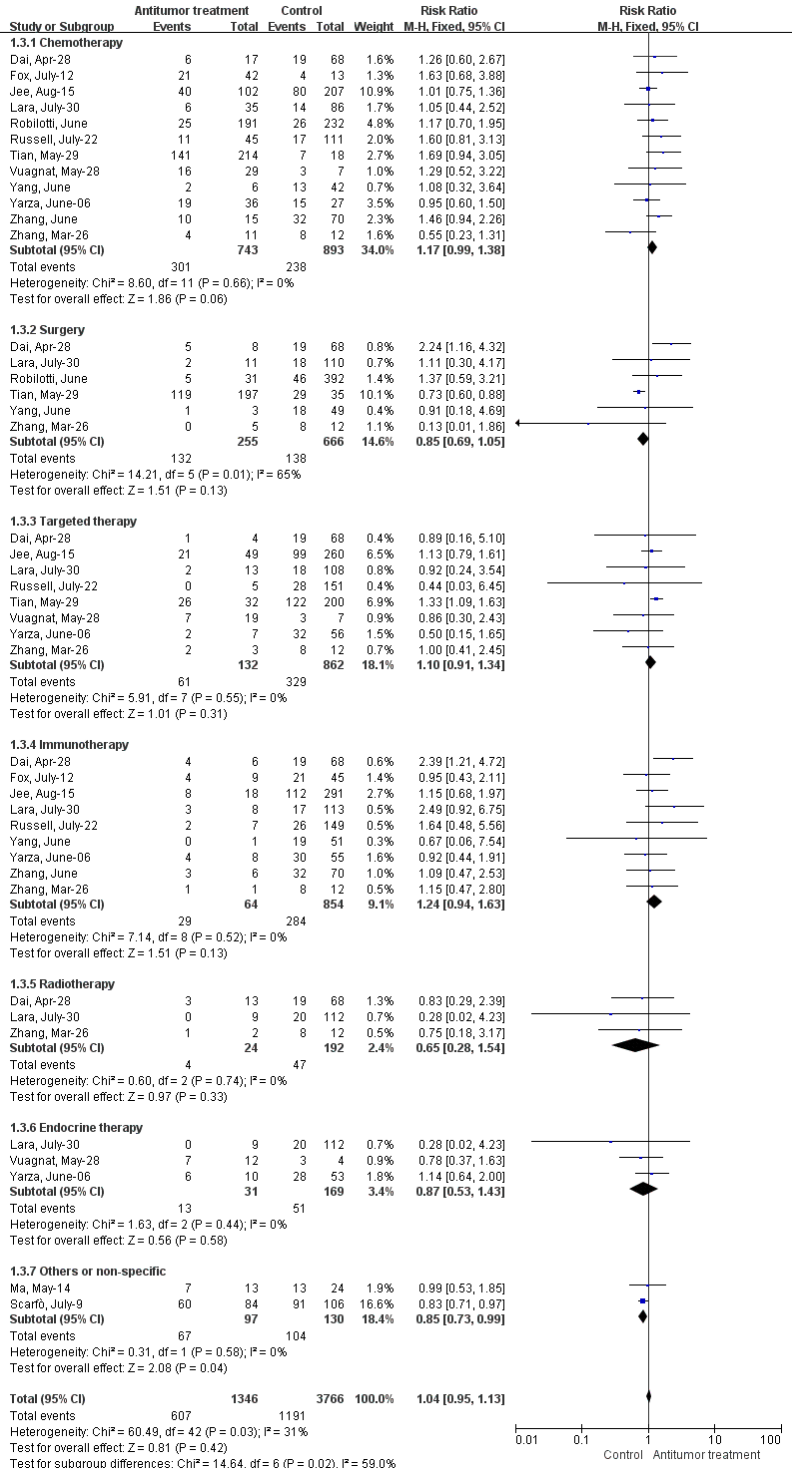
Forest plot for the association between antitumor treatments and the severe/critical rate in cancer patients with COVID‐19 using fixed‐effects model

The severe/critical rate was defined in accordance with the Diagnosis and Treatment Protocol for Novel Coronavirus Pneumonia released by the National Health Commission^15^ (Figure 3). Data analysis demonstrated that the antitumor treatments had no significant effects on the severe rates in COVID‐19‐infected patients with cancer (RR 1.04, 95% CI: 0.95–1.13, *I^2^*=31%, *p* = 0.42). Twelve studies provided data on the effects of chemotherapy, and no significant changes were observed between the two groups (RR 1.17, 95% CI: 0.99–1.38, *I^2^*=0%, *p* = 0.06). For other anticancer therapies, evaluations revealed that surgery (RR 0.85, 95% CI: 0.69–1.05, *I^2^*=65%, *p* = 0.13), targeted therapy (RR 1.10, 95% CI: 0.91–1.34, *I^2^*=0%, *p* = 0.31), immunotherapy (RR 1.24, 95% CI: 0.94–1.63, *I^2^*=0%, *p* = 0.13), radiotherapy (RR 0.65, 95% CI: 0.28–1.54, *I^2^*=0%, *p* = 0.33), endocrine therapy (RR 0.87, 95% CI: 0.53–1.43, *I^2^*=0%, *p* = 0.58), and others (RR 0.85, 95% CI: 0.73–0.99, *I^2^*=0%, *p* = 0.04) exerted no effects on patients’ severe/critical rate.

The rate of respiratory support is another commonly observed outcome (Supplement 5 Figure S2). Chemotherapy had no effects on the respiratory rate in patients with cancer infected with COVID‐19 (RR 0.82, 95% CI: 0.43–1.58, *I^2^*=68%, *p* = 0.56), neither as targeted therapy (RR 0.74, 95% CI: 0.45–1.21, *I^2^*=0%, *p* = 0.23) or some other therapies (RR 0.81, 95% CI: 0.53–1.22, *I^2^*=0%, *p* = 0.31). Notably, we found a higher respiratory support rate in patients who received surgery than in those who did not (RR 1.87, 95% CI: 1.02–3.46, *I^2^*=0%, *p* = 0.04).

**FIGURE 4:**
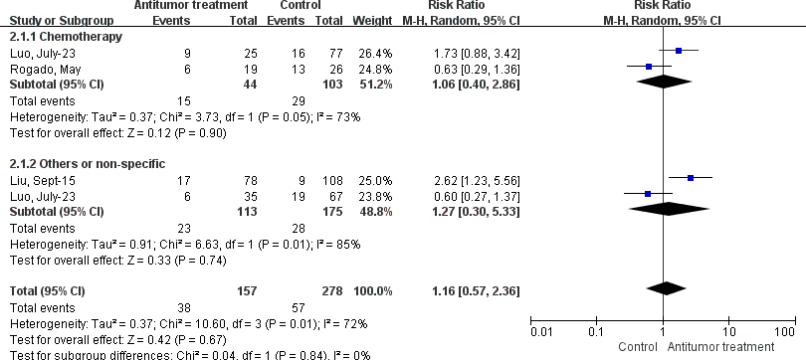
Forest plot for the association between antitumor treatments and the mortality rate in solid tumor patients with COVID‐19 using random‐effects model

**FIGURE 5:**
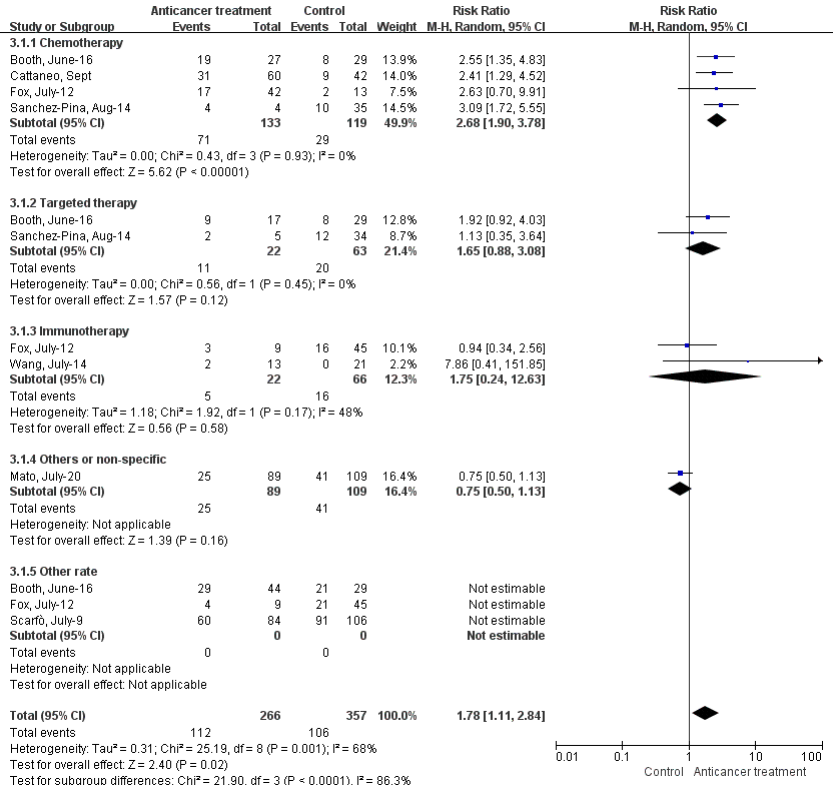
Forest plot for the association between antitumor treatments and the mortality rate in hematological malignancies patients with COVID‐19 using random‐effects model

In addition, we also analyzed the effects of anticancer treatments on solid tumors and hematological malignances. For patients co‐diagnosed with COVID‐19 and solid cancer, our data indicated that chemotherapy (RR 0.94, 95% CI: 0.68–1.32, *I^2^*=0%, *p* = 0.74), surgery (RR 0.58, 95% CI: 0.23–1.47, *I^2^*=18%, *p* = 0.25), targeted therapy (RR 0.76, 95% CI: 0.43–1.35, *I^2^*=0%, *p* = 0.35), immunotherapy (RR 1.19, 95% CI: 0.72–1.95, *I^2^*=0%, *p* = 0.49), radiotherapy (RR 0.47, 95% CI: 0.11–1.99, *I^2^*=0%, *p* = 0.30), endocrine therapy (RR 0.87, 95% CI: 0.53–1.43, *I^2^*=0%, *p* = 0.58), and other therapies (RR 0.99, 95% CI: 0.53–1.85, *p* = 0.99) had no effects on the severe rate (Supplement 5 Figure S3). In addition, there were no significant differences in the mortality when patients received chemotherapy (RR 1.06, 95% CI: 0.40–2.86, *I^2^*=73%, *p* = 0.90), or other treatments (RR 1.27, 95% CI: 0.30–5.33, *I^2^*=85%, *p* = 0.74) (Figure 4). With regard to patients suffering from COVID‐19 and hematological malignances, chemotherapy could markedly increase the mortality of these patients (RR 2.68, 95% CI: 1.90–3.78, *I^2^*=0%, *p* < 0.00001). However, no significant differences were observed when patients were treated with targeted therapy (RR 1.65, 95% CI: 0.88–3.08, *I^2^*=0%, *p* = 0.12), immunotherapy (RR 1.75, 95% CI: 0.24–12.63, *I^2^*=48%, *p* = 0.58), or other therapies (RR 0.75, 95% CI: 0.50–1.13, *p* = 0.16) (Figure 5).

## 4 DISCUSSION

This systematic review and meta‐analysis, in which a total of 5121 patients with cancer with COVID‐19 from 29 studies were included, is the largest study discussing the question to our knowledge. Our work did not suggest that the antitumor treatments would lead to poorer prognosis in patients with solid tumors diagnosed with COVID‐19. In contrast, patients with hematological malignancies are at higher risk of death if they receive chemotherapy in three months before the COVID‐19 diagnosis.

Since the first report by Liang et al,^5^ the treatment of cancer patients with COVID‐19 has been a hot topic. Cytotoxic chemotherapy, which can decrease the leukocyte count and lead to immunosuppressive status, has been reported to result in a high infection rate and poor prognosis.^38^, ^41^ The SARS‐CoV‐2 is more likely to trigger cytokine storm (CS) than other pulmonary infections. A CS will subsequently increase the incidence of the acute respiratory distress syndrome (ARDS), which has been observed in approximately 15% of cases.^42^ According to the study of Wan et al,^43^ IL‐6 was elevated significantly in the serum of severe cases, while CD4^+^ T cells, CD8^+^ T cells and natural killer cells were lower than those in mild cases. This may be explained by the reciprocal circle between the CS and the immunosuppressive status caused by chemotherapy and the cancer itself. In addition, chemotherapy for hematological malignancies will lead to a much higher rate of neutropenia and lymphocytopenia, which is considered a risk factor for mortality in patients with COVID‐19 in many studies.^44^ The elevated RR of the severe/critical rate in chemotherapy proves to support the theory. The adverse impact of cytotoxic chemotherapy on prognosis was also revealed in other viral infections.^45^, ^46^ Moreover, cytotoxic agents vary in their mechanisms and some agents were found to have anti‐CS effects,^47^ which may account for the high heterogeneity of chemotherapy. Targeted agents, which are highly selective to on co‐molecular targets, are generally thought to cause fewer side effects.^48^ The results of targeted therapy are similar to those of chemotherapy.

Patients receiving recent surgeries were reported to have a higher risk of viral infection and severe events,^8^ partially due to their frequent visits to hospitals and postoperative negative nitrogen balance. However, our results did not support this hypothesis. The higher rate of respiratory support in surgery patients may be explained by the routine use of postsurgical life support. In addition, the patients included in our meta‐analysis had distinct admission dates, which ranged from January to late May. Notably, their clinical management strategies have changed during this period.^49^, ^50^ Additionally, many elective operations were postponed or canceled while the remaining operations received special attention and care.

Radiotherapy has been confirmed to decrease lymphocytes and may lead to lymphopenia in some cases.^51^ Interestingly, our results showed that patients receiving radiotherapy tended to have a better prognosis than those not receiving radiotherapy, but a significant difference was not reached. Several scholars have supported the hypothesis that low‐dose radiation may mitigate the CS via pre‐consumption of immune reserves and a reduction in virus loading.^52^, ^53^ Hence, further investigations are warranted.

Immunotherapy represents another effective anticancer therapy with remarkable clinical benefits.There exist three major approaches to T cell‐based cancer immunotherapy, which are immune checkpoint blockade (ICI), adoptive cell transfer therapy, and active vaccination.^54^ Our results showed that immunotherapy had the highest risk among all anticancer treatments. The potential mechanism could be the activation of T cells by ICIs and a subsequent uncontrolled aberrant inflammatory response.^55^ Some researchers have now been working on a risk assessment scoring system to decide which patients with cancer could receive immunotherapy.^56^ To conclude, the prescription of immunotherapy should be used with extraordinary caution.

Although this meta‐analysis was carried out strictly conforming with the PRISMA, there were some limitations. The reliability of the results was to some extent weakened due to the lack of sufficient data. Some studies involved were single‐center and small‐sample studies, indicating the possibility of admission bias and sampling error. The ICU admission rate and the rate of respiratory support should be interpreted with caution, as they were highly related to the physicians’ experience. Due to the small sample size, chemotherapy had to be handled as a whole and subgroup analysis based on their individual pharmacological mechanism was difficult to perform. Furthermore, the effects of age, cancer type, and comorbidities were hard to evaluate. To conclude, the results of this systematic review should be interpreted with caution. However, the studies included were still the core of the evidence to date. A more persuasive study may re‐evaluate our conclusions.

This study was designed to provide physicians with more information about the safety of anticancer treatments in the COVID‐19 era. Bundles of studies have reported that the delay or cancelation of planned treatments during the pandemic might have a negative influence on patient prognosis.^57^, ^58^, ^59^ Although a 2‐month delay of treatment for stage I/II cancers was reported to be acceptable,^60^ the effect of delay in high‐stage cancers remains unclear, especially in patients older than 75.^61^ The clinical strategy for cancer management should be made based on the local medical capacity, the neighboring epidemic condition and the specific patient's condition. Telemedicine has been advocated by many experts in the follow‐up of non‐urgent cancer patients.^62^, ^63^ E‐visits, remote care management, and remote patient monitoring aids can be implemented using the social networks. For those at high risk of complications if their treatments are postponed, a systematic evaluation of the patient's conditions including RT‐PCR on nasopharyngeal swabs and thoracic CT is necessary.^64^ For those with oncologic emergencies, large lung masses, head and neck cancers and chemotherapy, or radiotherapy for high‐stage cancers,^57^ the active anticancer treatment should be received without any delay.

In conclusion, our results suggest that the chemotherapy for patients with hematological malignancies should be administrated with great caution. There was no stable evidence to confirm the adverse effect of any antitumor therapies in patients with solid tumors with COVID‐19. Some adverse tendencies have appeared in chemotherapy, surgery and immunotherapy, but none have reached a significant difference. Multicenter and prospective studies are needed to re‐evaluate our conclusions.

## Conflict of interest

All authors declare no competing interest.

## Authors’ contribution

Hanqing Liu and Dan Yang came up with the idea, searched the literature, selected the studies, extracted the data and draft the manuscript. Xinyue Chen and Zhihong Sun assessed the quality of each studies included. Yutong Zou provided the technical support and served as a senior reviewer in quality assessment. Chuang Chen served as a senior reviewer in study selection and data extraction. Shenrong Sun reviewed and polished the manuscript.

## Supporting information

Supplementary Material

Supplementary Material

Supplementary Material

Supplementary Material

Supplementary Material

## Data Availability

All data generated or analyzed during this study are included in this published article and supplement materials.
