# Supplementary material for: The effect of anticancer treatment on cancer patients with COVID‐19: A systematic review and meta‐analysis
Source: Cancer Med. 2020 Dec 31;10(3):1043–56. doi: 10.1002/cam4.3692 (PMC7897967; doi:10.1002/cam4.3692)
Supplement: Supplementary file 1 — Supplementary Material [file CAM4-10-1043-s001.docx]

| Supplement 1. PRISMA-P 2015 checklist: recommended items to include in a systematic review protocol ^a^ | | |
| --- | --- | --- |
| Section/topic | Item # | Checklist item |
| ADMINISTRATIVE INFORMATION | |  |
| Title |  |  |
| Identification | 1a | The report is a protocol of a systematic review. |
| Update | 1b | The protocol is not an update of a previous systematic review. |
| Registration | 2 | This meta-analysis has been registered in the PROSPERO database. (CRD42020200736) |
| Authors |  |  |
| Contact | 3a | Hanqing Liu, Renmin Hospital of Wuhan University, liuhqwhu@foxmail.com  Dan Yang, Renmin Hospital of Wuhan University, yangdanwhu@foxmail.com  Xinyue Chen, Renmin Hospital of Wuhan University, absalomchan@hotmail.com  Zhihong Sun, Renmin Hospital of Wuhan University, szh2608@126.com  Yutong Zou, Peking Union Medical College Hospital, 2472009606@qq.com  Chuang Chen, Renmin Hospital of Wuhan University, chenc2469@163.com  Shenrong Sun, corresponding author, Renmin Hospital of Wuhan University, sun137@sina.com,  Department of Thyroid and Breast Surgery, Renmin Hospital of Wuhan University, No. 99 Zhangzhidong Road, Wuhan 430060, Hubei, PR China |
| Contributions | 3b | Hanqing Liu and Dan Yang came up with the idea, searched the literature, selected the studies, extracted the data and draft the manuscript.  Xinyue Chen and Zhihong Sun assessed the quality of each studies included.  Yutong Zou provided the technical support and served as a senior reviewer in quality assessment.  Chuang Chen served as a senior reviewer in study selection and data extraction.  Shenrong Sun reviewed and polished the manuscript. |
| Amendments | 4 | No amendment of a previously completed or published protocol. |
| Support |  |  |
| Sources | 5a | National Natural Science Foundation of China (No. 81471781)  Fundamental Research Funds for the Central Universities (No. 2042019kf0229) |
| Sponsor | 5b | The Committee of National Natural Science Foundation of China  The Ministry of Education of P.R China and the Ministry of Finance of P.R China |
| Role of sponsor/ funder | 5c | The sponsor will cover the publishing fee. |
| INTRODUCTION |  |  |
| Rationale | 6 | COVID-19 has been pandemic and former studies have showed that cancer patients is likely to have worse diagnosis. |
| Objectives | 7 | We intend to explore how chemotherapy, targeted therapy, surgery, radiotherapy, immunotherapy and endocrine therapy impact on the mortality rate, ICU admission, rate of respiratory support, severity of the cancer patients diagnosed of COVID-19 compared to no antitumor treatment. |
| METHODS |  |  |
| Eligibility criteria | 8 | Inclusion: 1) study reporting the effect of any anti-tumor treatments on the mortality, ICU admission rate, rate of respiratory support or severe/critical rate in cancer patients diagnosed of COVID-19; 2) patients ≥ 18 years old; 3) the odd ratio (OR) or relative risk (RR) is extractable or relevant statistics are provided for calculation  Exclusion: 1) review, news, editorial, comment, guideline, clinical experience, basic research, study protocol and case report; 2) cancer patients <20 or couldn’t be separated from a whole patient group; 3) patients with other viral pneumonia were involved, such as SARS, MERS; 4) data derived from a same group of patients  Date: from 01-Dec-2019 to 23-Sept-2020  Language: no limitation. |
| Information sources | 9 | Web of Science (WOS), PubMed, Embase, Cochrane library, the China National Knowledge Infrastructure (CNKI) and the China Science and Technology Journal Database (VIP)  The lists of reference are also screening for any missing article. |
| Search strategy | 10 | Database: WOS, PubMed, Embase, Cochrane Library, the China National Knowledge Infrastructure, the China Science and Technology Journal Database  Date: from 01-Dec-2019 to 23-Sept-2020  Search sentence: (COVID-19 or SARS-CoV-2 or 2019-nCoV or coronavirus) and (tumor or carcinoma or cancer OR hematolog* OR haematolog* OR leukemia OR lymphoma OR myeloma)  Restriction:  1.no language limited  2.exclude review, case report, editorial, comment, animal research |
| Study records |  |  |
| Data management | 11a | Endnote (version 9.0) is adopted in the literature management. Excel is adopted in the data extraction and comparison. RevMan (5.3) and Stata (15.1) are used for data synthesis. |
| Selection process | 11b | Two independent reviewers carry out the literature screening with blindness to each other. The titles and abstracts are screened in first two rounds for efficiency. Then full articles are obtained for subsequent selection according to the criteria. Disagreements are resolved via consultation to a senior reviewer. |
| Data collection process | 11c | Two authors will extract the data independently and then cross-check their results. Disagreements will be resolved by consensus or consultation to a senior reviewer. We will contact the original author for any missing data. |
| Data items | 12 | The following data will be collected in a worksheet: first author, published date, country, study design, number of patients, number of females, median age, comorbidities, detection of COVID-19, cancer types, interpretation types and outcomes. The relative risks (RR) were obtained from the paper or calculated based on original statistics. |
| Outcomes and prioritization | 13 | Main outcome: mortality rate  Additional outcome: ICU admission rate, rate of respiratory support and severe/critical rate  The end-points should be measured in hospitals or medical institutions. The respiratory support referred to mechanical ventilation, facial mask and any other mechanical techniques improving the respiratory function. The definition of severe/critical rate should conform with the Diagnosis and Treatment Protocol for Novel Coronavirus Pneumonia released by National Health Commission. |
| Risk of bias in individual studies | 14 | The Newcastle-Ottawa Quality Assessment Scale for Cohort Studies will be adopted in the quality assessment. Eight questions in the scale are arrayed in three groups: patient selection, comparability and outcome reliability. The assessment will be done at study level. Two reviewers will assess the risk of bias independently with blindness to each other. The disagreements will be settled by a third reviewer. |
| Data Synthesis | 15a | The minimum number for subgroup analyses is two. |
|  | 15b | The data synthesis will be performed on RevMan and Stata. Relative risks and their 95% confidence interval (CI) will be calculated to compare the mortality rate and other additional outcome between patients receiving antitumor treatment and the control patients. *P*-value 0.05 is deemed as statistically significant. The inconsistency index (*I²* statistic) and Cochran’s *Q* test will be adopted in the assessment of heterogeneity. If heterogeneity is low, a fixed-effects model will then be adopted in the estimation of the average effect and its precision. If heterogeneity is high, then the random model will be adopted. |
|  | 15c | The subgroup analyses will be performed in specific antitumor treatment. It is obvious that different antitumor treatment may cast different effect on the prognosis. The chemotherapy has the highest probability according to former studies. The tests of interaction between groups will be used. A subgroup analysis in solid tumor or hematological malignancies will also be peroformed in that some studies have showed patients with hematological malignancies tend to have poorer prognosis than those with solid tumors. |
|  | 15d | If quantitative synthesis is not appropriate, then we will tend to write a systematic review without meta-analysis. |
| Meta-bias(es) | 16 | The funnel-plot and the Egger’s test will be employed to estimate the publication bias. |
| Confidence in cumulative evidence | 17 | The GRADE system will be introduced to evaluate the confidence of the systematic review. |

PRISMA-P Preferred Reporting Items for Systematic review and Meta-Analysis Protocols.

^a^ It is strongly recommended that this checklist be read in conjunction with the PRISMA-P Explanation and Elaboration for important clarification on the items. Amendments to a review protocol should be tracked and dated. The copyright for PRISMA-P (including checklist) is held by the PRISMA-P Group and is distributed under a Creative Commons Attribution License 4.0.
