# Supplementary material for: The effect of anticancer treatment on cancer patients with COVID‐19: A systematic review and meta‐analysis
Source: Cancer Med. 2020 Dec 31;10(3):1043–56. doi: 10.1002/cam4.3692 (PMC7897967; doi:10.1002/cam4.3692)
Supplement: Supplementary file 3 — Supplementary Material [file CAM4-10-1043-s003.doc]

| Supplement 3. Risk of Bias Assessment | | | | | | | | | |
| --- | --- | --- | --- | --- | --- | --- | --- | --- | --- |
| **Study** | **Q1** | **Q2** | **Q3** | **Q4** | **Q5** | **Q6** | **Q7** | **Q8** | **Score** |
| Assaad, June-07 | b**🟑** | a**🟑** | a**🟑** | b | neither | b**🟑** | a**🟑** | a**🟑** | 6 |
| Booth, June-16 | a**🟑** | a**🟑** | a**🟑** | a**🟑** | neither | b**🟑** | a**🟑** | a**🟑** | 7 |
| Cattaneo, Sept | b**🟑** | a**🟑** | a**🟑** | b | neither | a**🟑** | a**🟑** | a**🟑** | 6 |
| Dai, Apr-28 | a**🟑** | a**🟑** | a**🟑** | b | neither | a**🟑** | a**🟑** | a**🟑** | 6 |
| Fox, July-12 | b**🟑** | a**🟑** | a**🟑** | b | neither | b**🟑** | a**🟑** | a**🟑** | 6 |
| Jee, Aug-15 | b**🟑** | a**🟑** | a**🟑** | b | neither | b**🟑** | a**🟑** | a**🟑** | 6 |
| Kuderer, May-28 | a**🟑** | a**🟑** | a**🟑** | a**🟑** | neither | b**🟑** | a**🟑** | a**🟑** | 7 |
| Lare, July-30 | b**🟑** | a**🟑** | a**🟑** | b | neither | b**🟑** | a**🟑** | a**🟑** | 6 |
| Lee, May-28 | a**🟑** | a**🟑** | a**🟑** | a**🟑** | neither | b**🟑** | a**🟑** | a**🟑** | 7 |
| Liu, Sept-15 | a**🟑** | a**🟑** | a**🟑** | b | neither | a**🟑** | a**🟑** | a**🟑** | 6 |
| Luo, July-23 | b**🟑** | a**🟑** | a**🟑** | b | neither | b**🟑** | a**🟑** | a**🟑** | 6 |
| Ma, May-14 | b**🟑** | a**🟑** | a**🟑** | b | neither | a**🟑** | a**🟑** | a**🟑** | 6 |
| Mato, July-20 | a**🟑** | a**🟑** | a**🟑** | b | a**🟑** | a**🟑** | a**🟑** | a**🟑** | 7 |
| Mehta, May-01 | b**🟑** | a**🟑** | a**🟑** | b | neither | b**🟑** | a**🟑** | a**🟑** | 6 |
| Pinato, July | b**🟑** | a**🟑** | a**🟑** | b | neither | b**🟑** | a**🟑** | a**🟑** | 6 |
| Robilotti, June | b**🟑** | a**🟑** | a**🟑** | b | neither | a**🟑** | a**🟑** | a**🟑** | 6 |
| Rogado, May | b**🟑** | a**🟑** | a**🟑** | b | neither | a**🟑** | a**🟑** | a**🟑** | 6 |
| Russell, July-22 | b**🟑** | a**🟑** | a**🟑** | b | neither | a**🟑** | a**🟑** | a**🟑** | 6 |
| Sanchez. Aug-14 | b**🟑** | a**🟑** | a**🟑** | b | neither | a**🟑** | a**🟑** | a**🟑** | 6 |
| Scarfò, July-9 | a**🟑** | a**🟑** | a**🟑** | b | neither | a**🟑** | a**🟑** | a**🟑** | 6 |
| Stroppa. May-14 | b**🟑** | a**🟑** | a**🟑** | b | neither | a**🟑** | a**🟑** | a**🟑** | 6 |
| Tian, May-29 | a**🟑** | a**🟑** | a**🟑** | b | a**🟑** | a**🟑** | a**🟑** | a**🟑** | 7 |
| Vuagnat, May-28 | b**🟑** | a**🟑** | a**🟑** | a**🟑** | neither | a**🟑** | a**🟑** | a**🟑** | 7 |
| Wang, July-14 | b**🟑** | a**🟑** | a**🟑** | b | neither | a**🟑** | a**🟑** | a**🟑** | 6 |
| Yang, June | b**🟑** | a**🟑** | a**🟑** | b | neither | a**🟑** | a**🟑** | a**🟑** | 6 |
| Yang, May-29 | a**🟑** | a**🟑** | a**🟑** | b | a**🟑** | a**🟑** | a**🟑** | a**🟑** | 7 |
| Yarza, June-06 | b**🟑** | a**🟑** | a**🟑** | a**🟑** | neither | a**🟑** | a**🟑** | a**🟑** | 7 |
| Zhang, June | a**🟑** | a**🟑** | a**🟑** | b | neither | a**🟑** | a**🟑** | a**🟑** | 6 |
| Zhang, Mar-26 | a**🟑** | a**🟑** | a**🟑** | b | neither | a**🟑** | a**🟑** | a**🟑** | 6 |
