# Supplementary material for: The effect of anticancer treatment on cancer patients with COVID‐19: A systematic review and meta‐analysis
Source: Cancer Med. 2020 Dec 31;10(3):1043–56. doi: 10.1002/cam4.3692 (PMC7897967; doi:10.1002/cam4.3692)
Supplement: Supplementary file 4 — Supplementary Material [file CAM4-10-1043-s004.docx]

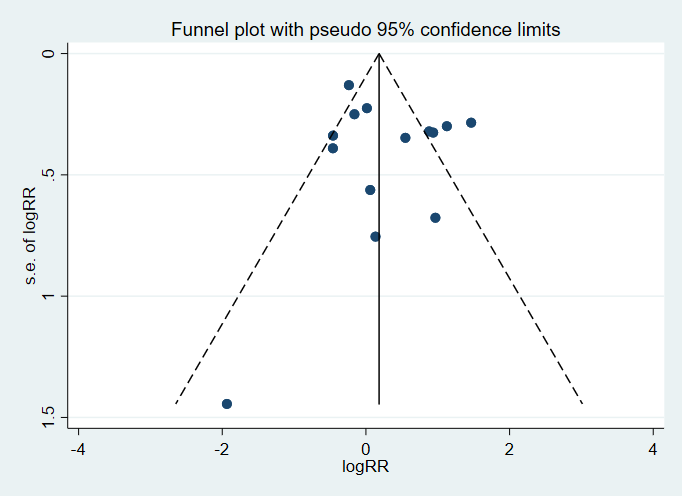

Supplement 4 Figure 1. Publication bias calculated with data in mortality & chemotherapy group


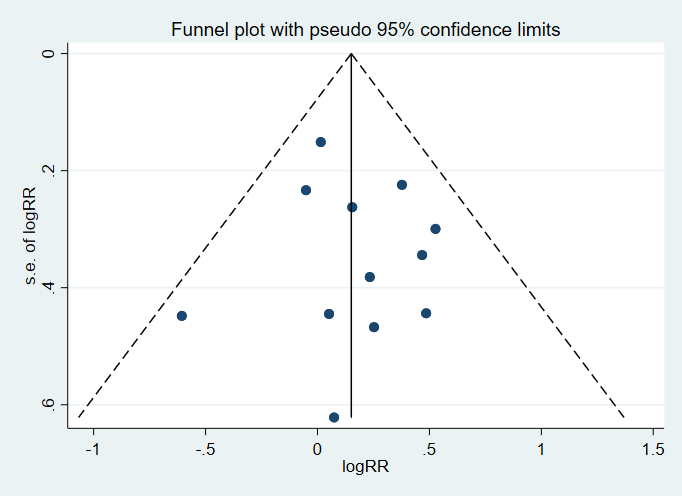

Supplement 4 Figure 2. Publication bias calculated with data in severe/critical rate & chemotherapy group
