# Supplementary material for: The effect of anticancer treatment on cancer patients with COVID‐19: A systematic review and meta‐analysis
Source: Cancer Med. 2020 Dec 31;10(3):1043–56. doi: 10.1002/cam4.3692 (PMC7897967; doi:10.1002/cam4.3692)
Supplement: Supplementary file 5 — Supplementary Material [file CAM4-10-1043-s005.docx]

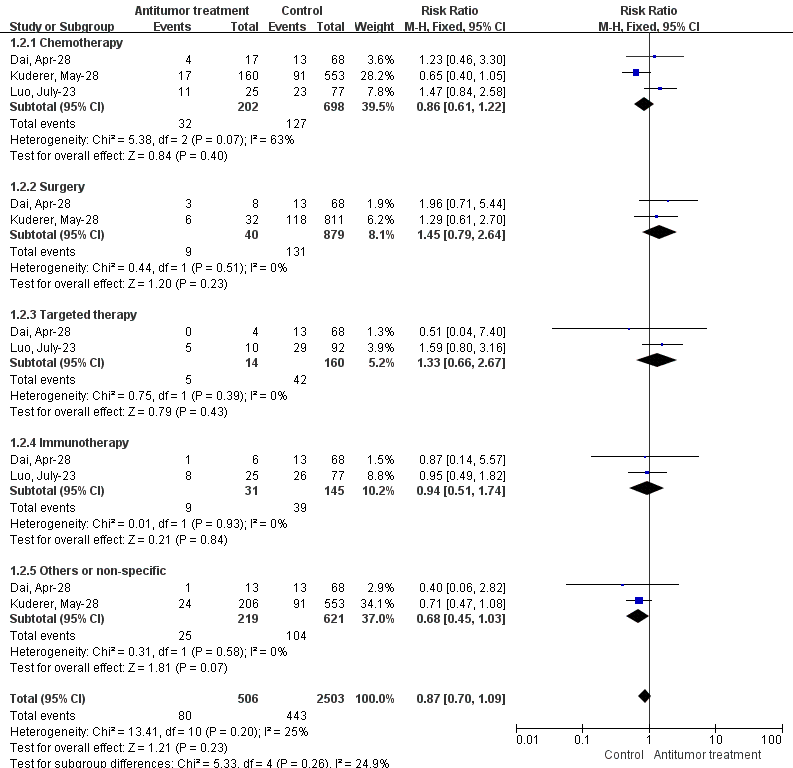


Supplement 5 Figure 1. Forest plot for the association between antitumor treatments and the ICU admission rate in cancer patients with COVID-19 using fixed-effects model.


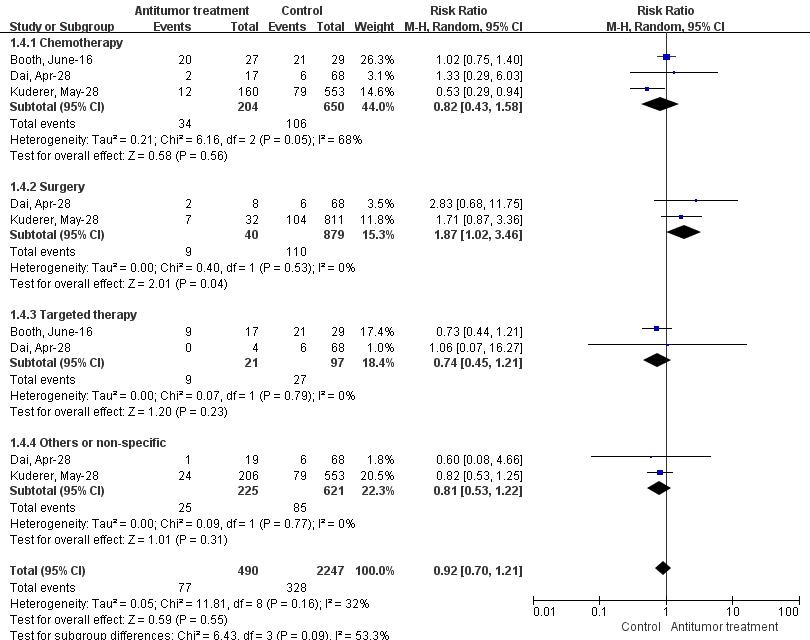


Supplement 5 Figure 2. Forest plot for the association between antitumor treatments and the rate of respiratory support in cancer patients with COVID-19 using random-effects model.


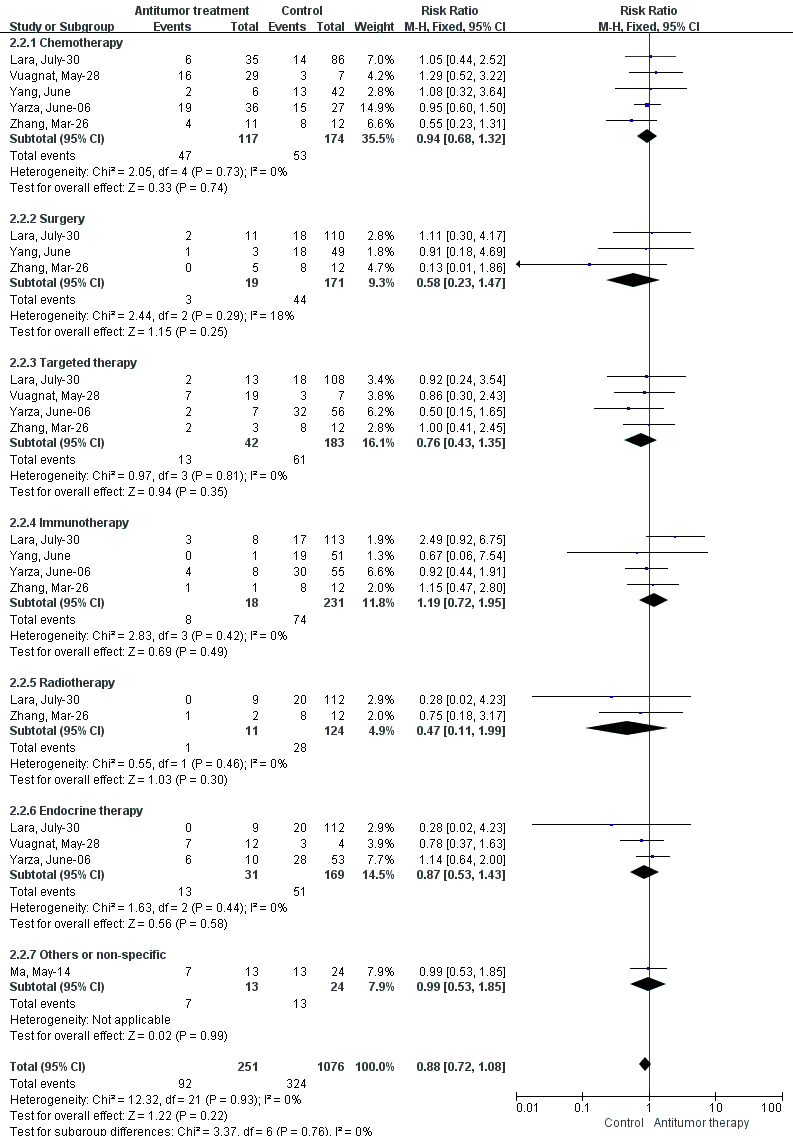


Supplement 5 Figure 3. Forest plot for the association between antitumor treatments and severe/critical rate in solid tumor patients with COVID-19 using fixed-effects model.
